# Role of Pancreatic Tumour-Derived Exosomes and Their Cargo in Pancreatic Cancer-Related Diabetes

**DOI:** 10.3390/ijms241210203

**Published:** 2023-06-15

**Authors:** Helen B. Binang, Chamini J. Perera, Minoti V. Apte

**Affiliations:** 1 Pancreatic Research Group, South Western Sydney Clinical Campuses, School of Clinical Medicine, Faculty of Medicine and Health, UNSW Sydney, Sydney, NSW 2052, Australia; h.binang@unsw.edu.au (H.B.B.); h.c.perera@unsw.edu.au (C.J.P.); 2 Ingham Institute for Applied Medical Research, Sydney, NSW 2170, Australia

**Keywords:** exosomes, pancreatic cancer-related diabetes, biomarkers, pancreatic cancer, pancreatic adenocarcinoma

## Abstract

One of the most common and deadly types of pancreatic cancer (PC) is pancreatic ductal adenocarcinoma (PDAC), with most patients succumbing to the disease within one year of diagnosis. Current detection strategies do not address asymptomatic PC; therefore, patients are diagnosed at an advanced stage when curative treatment is often no longer possible. In order to detect PC in asymptomatic patients earlier, the risk factors that could serve as reliable markers need to be examined. Diabetic mellitus (DM) is a significant risk factor for this malignancy and can be both a cause and consequence of PC. Typically, DM caused by PC is known as new-onset, pancreatogenic, pancreoprivic, or pancreatic cancer-related diabetes (PCRD). Although PCRD is quite distinct from type 2 DM (T2DM), there are currently no biomarkers that differentiate PCRD from T2DM. To identify such biomarkers, a better understanding of the mechanisms mediating PCRD is essential. To this end, there has been a growing research interest in recent years to elucidate the role of tumour-derived exosomes and their cargo in the pathogenesis of PCRD. Exosomes derived from tumours can be recognized for their specificity because they reflect the characteristics of their parent cells and are important in intercellular communication. Their cargo consists of proteins, lipids, and nucleic acids, which can be transferred to and alter the behaviour of recipient cells. This review provides a concise overview of current knowledge regarding tumour-derived exosomes and their cargo in PCRD and discusses the potential areas worthy of further study.

## 1. Introduction

Pancreatic cancer (PC) is the seventh leading cause of cancer-related deaths in the world, with the most common type, comprising >90% of PC cases, taking the form of pancreatic ductal adenocarcinoma (PDAC) [1,2,3]. The incidence of PC is around 45 out of every 100,000 people, and currently, the 5-year survival rate is dismal, hovering around 11.5% [4]. Deaths from PC are on the rise. For example, in Australia, PC was the fourth most common cause of cancer deaths in 2020 and the third most common cause of death from cancer in 2022 [4]. The disease is often silent in nature, and symptoms only manifest after it has progressed to an advanced stage. Indeed, the symptoms of PC are often non-specific, ranging from vague abdominal pain, general malaise, nausea and vomiting, poor appetite, and weight loss. The poor clinical outcomes in this disease are a result of several factors, including late diagnosis, early distant metastasis, resistance to most conventional treatment options, and a dense tumour microenvironment that inhibits the penetration of drugs into the tumour and actively facilitates tumour progression via a range of mechanisms [5].

The major risk factors for PC include older age, sex (high prevalence in men), smoking, a history of chronic pancreatitis (CP), gene mutations, obesity, and diabetes mellitus (DM) [6]. Obesity and CP are also associated with metabolic disorders such as insulin resistance and glucose intolerance, which are characteristics of diabetes [7,8]. However, as CP is a known cause of diabetes, whether the metabolic defects seen in obesity are causes or effects of diabetes and/or PC remains to be clearly established [9,10].

### 1.1. Pancreatic Cancer and Diabetes

Diabetes mellitus is a heterogeneous metabolic disorder that is characterized by the presence of hyperglycaemia due to the impairment of insulin secretion and/or defective insulin action leading to peripheral insulin resistance. It is a major public health problem that affects over 462 million people worldwide [11]. Its incidence is predicted to reach 7079 per 100,000 by 2030 and 7862 per 100,000 by 2040 [12]. It is associated with increased risks for stroke, blindness, amputations, heart disease, kidney disease and cancer. DM can be classified into three main categories, namely type 1 DM (T1DM), type 2 DM (T2DM), and gestational diabetes. A form of DM associated with diseases of the exocrine pancreas is known as type 3c DM (T3cDM) [13]. DM can be both a cause and a consequence of PC [14].

### 1.2. Pancreatic Cancer-Related Diabetes (PCRD)

A subset of PC patients reported being diagnosed with DM within 3–5 years before their diagnosis of PC. This recent onset of diabetes has also been termed pancreatic cancer-related diabetes (PCRD) and may be a harbinger of PC [9]. PCRD may be caused by mediators of β cell dysfunctions and peripheral insulin resistance, the interaction between PC and adipose tissue, the control of DM-associated PC pathway genes by miRNAs and the accelerated transforming growth factor β (TGF-β) signalling due to the increased secretion of TGF-β, causing the depletion of β cells [15,16,17,18,19,20,21,22,23,24,25,26,27,28]. There are distinct differences between the clinical picture of PCRD and that of type 2 DM (T2DM). Unlike T2DM, PCRD is characterized by weight loss and often normal insulin levels. Notably, the resolution of diabetes occurs after pancreatic tumour resection [29,30,31] (Table 1).

The underlying association between PC and DM is complicated due to the presence of a bidirectional link. The ability to distinguish DM caused by PC from T2DM is critical for recognizing that the new onset of DM may indicate a developing PC. Therefore, identifying novel, clinically applicable biomarkers of high sensitivity and specificity in the setting of PCRD may lead to earlier PC detection and subsequently improved therapeutic outcomes [32,33]. It is possible that developing cancer from its earliest stage, called pancreatic intraepithelial neoplasm (PanIN), secrete factors that are carried within exosomes (a type of extracellular vesicle, EV) to impair the function of islets and other peripheral cells, leading to decreased insulin secretion and/or peripheral insulin resistance. Thus, studies of tumour-derived exosomes and their cargo have recently become a focus of research in order to understand their role in PCRD.

Since the best-studied EVs in the literature are exosomes, this review focused on the possible role of exosomes as mediators of PCRD. We summarized the recent work on exosomes in relation to PC as well as DM and the specific roles of pancreatic tumour-derived exosomes in PCRD development. By unveiling the putative adverse influence of PC on systemic glucose metabolism resulting in PCRD, potential biomarkers could be uncovered to improve the early diagnosis and treatment of PC.

## 2. Exosomes

Exosomes are part of a family of membrane-derived particles that are collectively known as EVs. They facilitate intercellular communication (in health as well as in diseased states) via their cargo which consists of a mixture of bioactive molecules such as nucleic acids (mRNAs, miRNAs, DNA), lipids and proteins, which can be transported to the targeted cells/tissues via the blood or lymph circulation [34]. Based on their molecular sizes and mode of formation, EVs can be classified as apoptotic bodies, microvesicles and exosomes [35].

### 2.1. Exosome Formation and Characterization

**Figure 1:**
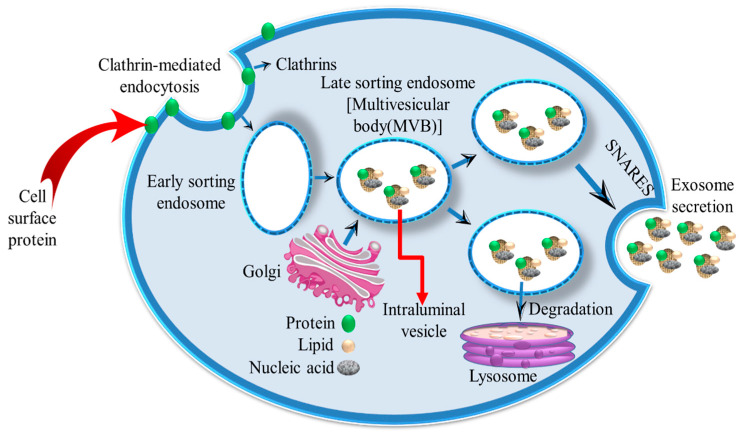
Biogenesis and the secretion of exosomes. Exosome biosynthesis begins during the maturation of the early sorting endosome into the late sorting endosome or multivesicular body (MVB). Exosome precursors known as intraluminal vesicles (ILVs) are contained within MVBs. MVBs can either be degraded (via fusion with lysosomes) or transported to the plasma membrane. Then, ILVs are released as exosomes into body fluids during the fusion of MVBs with the plasma membrane.

Exosomes are small Evs (sEVs) that are contained within multivesicular bodies (MVBs) and are released when the MVBs fuse with the plasma membrane (Figure 1). Exosome biogenesis begins when the early-sorting endosome matures into a late-sorting endosome or MVB [36]. The MVBs contain intraluminal vesicles (ILVs), which are the exosome precursors. MVBs can either be degraded (via fusion with lysosomes) or transported to the plasma membrane for exosome release. The fusion of MVBs with the plasma membrane results in ILVs being released as exosomes into body fluids.

Exosomes are cup-shaped, cell-specific, small double-membraned EVs that are heterogeneous in size and range from 30 to 150 nm; they are produced by different cell types and can be found in all biological fluids. They are identified based on their size, density, morphology, and the presence of certain surface markers. The common surface markers of exosomes are tetraspanins (CD63, CD81 and CD9), fusion proteins (Flotilin, Annexins, GTPases), endosome-associated proteins (Alix, TSG101) and heat shock proteins (Hsp70, Hsp90) [37].

### 2.2. Uptake of Exosomes by Recipient Cells

**Figure 2:**
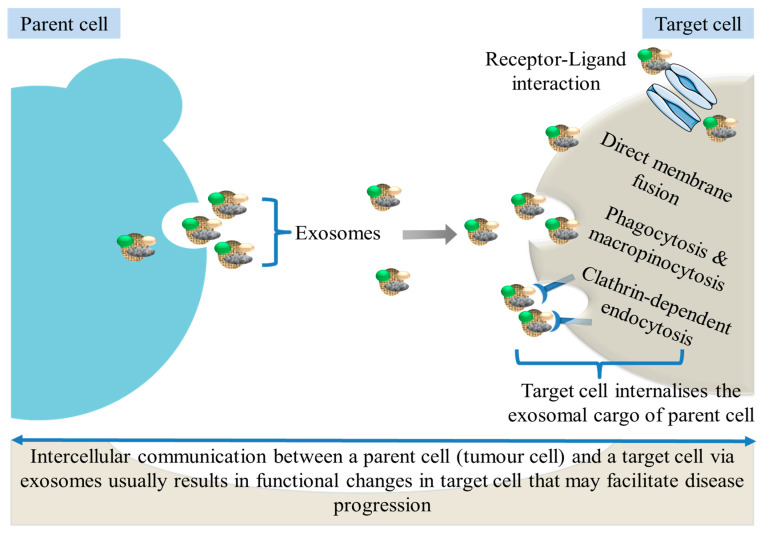
Exosome release and uptake. Exosomes released by parent cells (tumour cells) can be taken up via the receptors on recipient cells or through mechanisms such as direct membrane fusion, phagocytosis, macropinocytosis and clathrin-dependent endocytosis. Exosomal cargo: green = proteins, yellow = lipids, grey = nucleic acids.

Generally, cells may take up exosomes through a variety of endocytic pathways, namely: clathrin-independent pathways such as direct membrane fusion, macropinocytosis, phagocytosis or via clathrin-dependent endocytosis (Figure 2). Other pathways of exosome uptake include caveolin-mediated uptake and lipid raft-mediated internalization. The mechanism of this uptake adopted by target cells for a given exosome may depend on proteins and glycoproteins, which can be found on the surface of both the exosome and the target cell [38]. Upon being taken up by target cells, exosomes may influence cell function via the release of cargo molecules which can activate intracellular signalling. Exosomes and their cargo are involved in the pathogenic processes of both PC [39,40,41] and DM [42,43].

As noted earlier, the mechanisms of PCRD may be centred around the influence of pancreatic tumour-derived exosomes or the ability of β cells to produce insulin and that of other cells to produce, take up or utilize glucose.

## 3. Pancreatic Cancer-Derived Exosomes

The use of tumour-derived exosomes as liquid biopsies in cancer management is currently an attractive area of investigation with promising results [44]. This is because obtaining samples from patients for exosome isolation is minimally invasive as exosomes are present in body fluids such as blood, saliva, ascites or urine. In addition, exosomes are highly stable and specific, and the exosome cargo, namely, proteins, lipids and nucleic acids are highly reflective of parental cells. As such, it is possible to understand the behaviour of certain cells by examining the cargo contained in their exosomes. Exosomes produced under pathological conditions often exhibit tendencies that are different from those obtainable under normal conditions. In cancers, exosomes produced by tumour cells contain molecules that are cell-specific, and these molecules can be used to differentiate tumour cells from normal cells and hence, cancer patients from healthy individuals [45]. However, the processes surrounding these differences in the exosome composition among cells are not yet known, and the means by which these processes are regulated remains to be fully elucidated.

## 4. Role of Pancreatic Tumour-Derived Exosomes in PCRD

Exosomes originating from PC cells can transport cargo molecules to different cell types, ultimately affecting disease progression. PC-derived exosomes can regulate angiogenesis, deliver tissue factor (TF) promoting cancer-associated thrombosis, regulate immune functions, promote tumour growth, invasion and metastasis, confer chemoresistance, and regulate pancreatic functions. The role of tumour cell-derived exosomes in pancreatic cancer per se has been reported in detail within previous publications [46,47]. Here, we discuss the current knowledge about the role of PC-derived exosomes and their cargo in PCRD.

### 4.1. Exosomal Protein Cargo and PCRD

Studies have shown that certain proteins contained within exosomes derived from pancreatic tumour cells play key roles in the development of PCRD: the most important being adrenomedullin (AM).

#### Adrenomedullin

This component of PC-derived exosomes is a multifunctional vasoactive peptide that has been implicated in inflammation and sepsis and is overexpressed in PC at both the mRNA and protein levels [48]. High expressions of AM can cause decreased β-cell insulin secretion, as evidenced by studies showing that when β cell lines have been treated with glucose followed by exposure to AM, glucose-stimulated insulin secretion was inhibited [17]. This effect is more pronounced in β-cell lines or islets when isolated from mice but has also been rectified following the small hairpin RNA (shRNA)-mediated knockdown of AM in PC cells. Another study showed that PC-derived exosomes containing AM and carbohydrate antigen 19-9 (CA19-9, also known as sialyl Lewis-a (sLea)—a widely used diagnostic and prognostic marker for PC [49]—readily entered β cells through caveolin-mediated endocytosis or macropinocytosis, and induced cyclic adenosine monophosphate (cAMP) production which is usually associated with increased insulin secretion [42]. However, low insulin secretion was also observed in this study despite the overexpression of cAMP; this points to the induction of endoplasmic reticulum (ER) stress in a process independent of cAMP. Interestingly, the ER stress markers immunoglobulin heavy chain binding protein (BiP) and C/EBP homologous protein (CHOP) were overexpressed, causing an increase in the unfolded protein response (UPR) and overwhelming ER stress in the β cells. This resulted in the upregulation of oxidative stress as indicated by the high expressions of reactive oxygen species (ROS) and reactive nitrogen species (RNS), leading to marked β cell dysfunction. Hence, one of the ways in which PC causes paraneoplastic β cell dysfunction may be through the shedding of AM(+)/CA19-9(+) exosomes into the circulation that inhibit insulin secretion in PCRD; this is likely through the AM-induced failure of UPR and ER stress [42].

**Figure 3:**
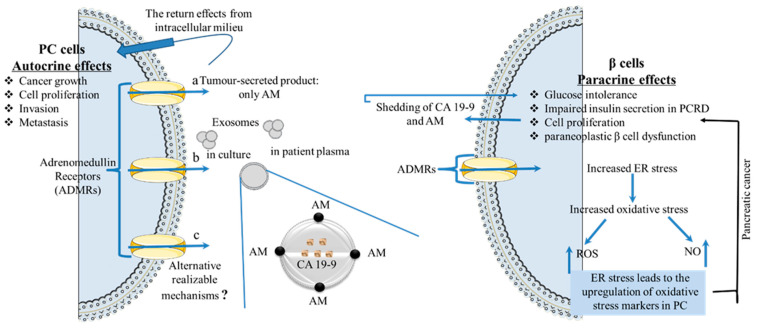
Integrated schematic workflow elucidating the potential mechanism of action of pancreatic cancer (PC)-derived adrenomedullin (AM) on PC cells and β cells. (a) AM may be present in tumour cell-derived exosomes either alone or (b) Together with CA 19-9, (c) Alternative channels for AM secretion are yet to be brought to scientific limelight. AM may then further act upon PC cells in an autocrine manner and on β cells in a paracrine manner with a variety of pro-tumorigenic and pro-diabetogenic effects, respectively. ? = unknown.

Studies in mice have also shown that AM and its receptor components—the calcitonin receptor-like receptor (CRLR) and receptor activity modifying proteins (RAMPs)—are present in pancreatic islets and can be co-localized with insulin [50]. Another AM receptor (ADMR) found on β cells acted as a peptide mediator for AM and was carried within PC-derived exosomes. The interaction of AM with this receptor caused impaired insulin secretion, as seen in PCRD, to initiate the dysfunction of the recipient cells [42]. Interestingly, the ADMR blockade abrogated the inhibitory effect of exosomes during insulin secretion [51] (Figure 3).

Aggarwal et al. demonstrated that mice bearing pancreatic tumours formed from PC cell lines and overexpressing AM were significantly glucose intolerant compared to control mice bearing tumours formed from PC cells transfected with the control vector [17]. In addition to inducing glucose intolerance, PC-derived exosomal AM was found to promote lipolysis in murine and human adipocytes. AM interacted with its receptor on the adipocytes, activated p38, extracellular signal-regulated kinase 1/2 (ERK1/2) and mitogen-activated protein kinases (MAPKs) and promoted lipolysis through the phosphorylation of hormone-sensitive lipase [51]. Hence, AM in pancreatic tumour exosomes may play a role in PCRD not only via its effects on the β cell function but also by inducing lipolysis, which is a major contributor to insulin resistance.

Studies have also identified other proteins which have been speculated to be involved in the pathogenesis of PCRD and which have not yet been identified or characterized as exosomal cargo. These include:

1. The PC-derived S-100A8 N-terminal peptide: a diabetogenic agent with reduced glucose consumption and lactate production by myoblasts in vitro [52]. This peptide also hindered the growth of myoblasts by inhibiting myotubular differentiation and increasing caspase-3 activation in cultured C2C12 myoblasts. S-100A8 is, therefore, suggested to be a cause of hyperglycemia because it impairs glucose catabolism in myoblasts and is speculated to be a promising biomarker for the diagnosis of PCRD. In addition, S-100A8 N-terminal alters β-cell insulin secretion by inhibiting glucose-stimulated insulin exocytosis (early response), which is characteristic of PCRD [53].
2. The insulin-like growth factor 1 (IGF-1) and Insulin-like growth factor-binding protein-2 (IGFBP-2) are biomarkers of pancreatic diseases, especially CP and PC. Since the expression of IGF-1 in CP and DM was elevated compared with that in PC and DM, IGF-1 may be an indicator that signals whether pancreatic diabetes is from CP or PC [54].
3. In the serum taken up to 4 years before a PC diagnosis, circulating Thrombospondin 1 (TSP-1) levels were found to be significantly reduced up to 24 months prior to diagnosis. Low serum TSP-1 levels in PC patients were associated with DM. Interestingly, TSP1 levels in PC patients with DM were lower compared to patients with T2DM alone. TSP-1 was also decreased in PC patients, compared to the healthy controls and patients with benign biliary obstruction at clinical diagnosis. Not only did circulating TSP-1 levels decrease up to 24 months before the diagnosis of PC, but a combination of TSP-1 and CA19-9 also produced a diagnostic capacity that significantly outperformed both markers alone. Decreased TSP-1 levels may be an indication of PCRD, and early PC detection strategies could include exploring the clinical relevance of TSP-1.
4. The vascular noninflammatory molecule 1 (Vanin 1/VNN1) is a pantetheinase that is anchored to the extracellular membrane of epithelial and myeloid cells. It belongs to an enzymatic pathway and causes oxidative stress, inflammation and cell migration and is suggested to be a biomarker for certain malignancies, including systemic lupus erythematosus and type 1 diabetic nephropathy [55,56]. It was overexpressed in PC and inhibited the growth of insulin-secreting cell lines (β-TC-6 and INS-1) when they were treated with conditioned media derived from PC cell lines. This loss of cell viability was even greater when the cells were exposed to conditioned media from PC cells transfected with a VNN1-overexpressing vector, indicating that the high expression of VNN1 causes injury to paraneoplastic insulin-secreting cells. The high expression of VNN1 also altered the expressions of oxidative stress-related factors, including the downregulation of the anti-oxidative stress/anticancer peroxisome-proliferator activated receptor γ (PPAR-γ), the upregulation of cysteamine which is a product of VNN1, the downregulation of the antioxidant glutathione (GSH) and upregulation of ROS. Hence, the overexpression of VNN1 in PC cells modulated the viability and function of β cells by promoting oxidative stress in the β cells’ microenvironment [57]. Additionally, VNN1, when used in combination with the matrix metalloproteinase 9 (MMP9), could be a more effective blood biomarker panel for the discrimination of PCRD from T2DM [58].

### 4.2. Exosomal Lipid Cargo and PCRD

There is currently no experimental evidence to show the role of exosomal lipids in PCRD. However, adiponectin, a hormone, which is mainly produced by adipocytes (fat cells), is known to increase insulin sensitivity and regulate peripheral glucose levels and fatty acid metabolism [59]. Additionally, the combination of the adiponectin and interleukin-1 receptor antagonist (IL-1Ra), which was elevated in PC patients with DM compared to those with T2DM and the healthy controls showed strong diagnostic potential for the distinction of PCRD from T2DM [60]. Tumour cells produce a range of interleukins; hence, IL1-R antagonist levels in PC cell-derived exosomes could be worthy of study, particularly in screening for PC in individuals newly diagnosed with T2DM.

### 4.3. Exosomal RNA Cargo and PCRD

The two types of ribonucleic acid (RNA)—coding and non-coding RNA—are both essential for gene expression. Altered processing or the activity of RNA, whether coding or non-coding, is a hallmark of cancers [61]. However, over the years, there has been accumulated evidence regarding the role of the aberrant expression of non-coding RNAs (ncRNAs) in many cancers [62,63,64,65], including PC and also in other diseases such as DM. These ncRNAs play important roles in epigenetic processes, transcription and post-transcriptional regulation and can, thus, affect the growth, migration and invasion of cells. The role of ncRNAs in cancers (including PC) and other human diseases (including DM) has been reviewed elsewhere [66,67].

Examples of ncRNAs include small RNAs such as microRNAs (miRNAs), small interfering RNAs (siRNAs), Piwi-interacting RNAs (piRNAs), small nucleolar RNAs (snoRNAs), small nuclear RNAs (snRNAs), small cajal body-specific RNAs (scaRNAs), as well as extracellular RNAs (exRNAs), long non-coding RNAs (lncRNAs), long intergenic non-coding RNAs (lincRNAs), circular RNAs (cirRNAs), transfer RNAs (tRNAs), messenger RNAs (mRNAs) and ribosomal RNAs (rRNAs). Of these non-coding RNAs, the best studied with regard to exosmal cargo and PCRD are miRNAs, with little information available about other exosomal non-coding RNAs.

#### Exosomal miRNAs and PCRD

Several studies indicate that microRNA (miRNA) fragments have diagnostic, predictive and therapeutic usefulness in different cancer types, including PC. With regard to PCRD, the effects of exosomes released by PC cells (MIA PaCa-2) on the intestinal secretin tumour cell line (STC-1), which expresses many features of normal enteroendocrine cells, were explored. Normally, enteroendocrine cells are responsible for the production of a glucose-dependent insulinotropic peptide (GIP) and glucagon-like peptide-1 (GLP-1), both of which function in maintaining glucose homeostasis by enhancing insulin secretion and sensitivity [68]. Zhang et al. found that the treatment of STC-1 cells with exosomes derived from PC cells resulted in the downregulation of GLP-1 and GIP [69]. These authors reported that this effect was mediated by miR-6796-3p, miR-6763-5p, miR-4750-3p and miR-197-3p within the exosomes, as evidenced by the fact that the inhibition of the expression of these miRNAs in the parental tumour cells (MiaPaCa-2) reversed the downregulation of GLP-1 and GIP by downregulating the post-translational proprotein convertase subtilisin/kexin type 1/3 (PCSK1/3) levels in STC-1 cells [69].

In a study by Su et al., exosomal miR-19a from PC cells was found to downregulate insulin secretion in mouse insulinoma 6 cells (MIN6) and primary islets by targeting Neurod1: the validated gene involved in insulin secretion [70]. cAMP, which potentiates insulin secretion, is produced by the cell membrane protein adenylyl cyclase (ADCY) and ATP, and Adcy1 is a member of the ADCY family, which is activated by extracellular Ca^2+^ in MIN6 cells [71]. The binding of miR-19a to Neurod1 caused the downregulation of cAMP and Ca^2+^ expression in β cells, leading to decreased insulin secretion [70]. Another independent report demonstrated that PC-derived exosomal miR-19a targets Adcy1 and Epac2 were an exchange protein directly activated by cAMP 2. Both these factors are involved in insulin secretion, thereby inducing β cell dysfunction in PCRD [43]. This indicates that signal changes between PC cells and β cells via exosomes might be important in the pathogenesis of PCRD and provide a possible basis for the application of exosomal miR-19a in a PC screening strategy.

Regarding insulin resistance, PC-derived exosomes were found to readily enter C2C12 myotubes, causing lipidosis and the inhibition of glucose uptake by impairing glucose transporter type 4 (GLUT4) trafficking and preserving forkhead box protein O1 (FOXO1) nuclear exclusion [72]. FOXO1 is a nuclear transcription factor whose expression can be induced by the presence of insulin; therefore, it can be overexpressed in skeletal muscles in energy-deprived states such as diabetes and is a key target of the PI3K/Akt signalling pathway [73]. Once activated/phosphorylated by activated Akt, FOXO1 could be excluded from the nucleus, resulting in the repression of transcription and muscle insulin resistance. FOXO1 in PC-derived exosomes inhibited GLUT4 expression as well as insulin and PI3K/Akt signalling [72]. Interestingly, miRNAs were found to be upregulated in PC-derived exosomes including miR-666-3p, miR-540-3p, miR-125b-5p and miR-450b-3p promoted FOXO1 expression, while miR-883b-5p, 666-3p, miR-450b-3p and miR-151-3p downregulated GLUT4 expression. Therefore, PC-derived exosomal miRNAs were found to be capable of inducing the insulin resistance of skeletal muscle cells through insulin and PI3K/Akt/FoxO1 signalling pathways [72].

Another miRNA that could play a role in insulin resistance is miRNA-let-7b-5p, which has been shown to interact directly with SLC6A15: a gene associated with the insulin resistance of metabolic disorders. miRNA-let-7b-5p inhibits SLC6A15 expression via its effects on the SLC6A15 3′-untranslated region (3′-UTR) and is thought to promote insulin resistance in C2C12 myotube cells [74].

Studies have also shown a few other non-coding RNAs, including miRNAs and long non-coding RNAs (lncRNAs), which may be involved in the pathogenesis of PCRD but are yet to be investigated in exosomes. For instance, a panel of six serum miRNAs (miR-483-5p, miR-19a, miR-29a, miR-20a, miR-24, miR-25) upregulated in PCRD was developed as a biomarker for PCRD, with a significant diagnostic efficacy for the discrimination of PCRD from non-cancer new-onset T2DM, signifying that they may be involved in PCRD development [75].

Another study reported the association of seven lncRNAs and two miRNAs with prognosis in patients with PCRD. The lncRNAs included (i) the DiGeorge syndrome critical region gene 9 (DGCR9), (ii) FLJ33360, (iii) H19, (iv) HOX Transcript Antisense RNA (HOTAIR), (v) KIAA0125, (vi) Small Cajal Body-Specific RNA 2 (SCARNA2) and (vii) Urothelial Cancer Associated 1 (UCA1). The two miRNAs were (i) hsa-miR-214 and (ii) hsa-miR-429. Notably, hsa-miR-214 was predicted to target the lncRNA HOTAIR and its downstream mRNA target Coiled-Coil Domain Containing 33 (CCDC33). Similarly, hsa-miR-429 could target Cat Eye Syndrome Chromosome Region, Candidate 7 (CECR7) and the Cytotoxic T-Lymphocyte Associated Protein 4 (CTLA4) downstream, suggesting that these RNAs regulate each other post-transcriptionally as they compete when binding to the miRNAs (a phenomenon known as the competing endogenous RNA network). ‘HOTAIR-hsa-miR-214-CCDC33’ and ‘CECR7-hsa-miR-429-CTLA4’ regulations might be two important mechanisms for PC progression, requiring further investigation. HOTAIR, CECR7, UCA1, hsa-miR-214, hsa-miR-429, CCDC33 and CTLA4 were also highlighted as potential diagnostic biomarkers [76], though these were not characterized in exosomes.

## 5. Tumour-Derived Exosomal Cargo and Pancreatic Cancer

Additionally, worthy of note is the fact that exosomes derived from pancreatic tumours can exacerbate the aggressiveness of PC mainly via their protein and RNA cargo components [77]. By taking up exosomes/exosomal cargo released by highly malignant PC cell lines, moderately malignant PC cell lines acquire increased proliferative, migratory, and invasive abilities. For example, the zinc/iron-regulated transporter-like protein 4 (ZIP4) is a protein upregulated in the highly malignant PC cell line—PC-1.0 that promotes tumour growth both in vitro and in vivo [78]. When less malignant PC cell lines such as PC-1 take up ZIP4-containing exosomes derived from PC-1.0 cells, the recipient cells become more aggressive, suggesting the role of exosomal ZIP4 in PC progression. Exosomal proteins have also been reported to be associated with the resistance of PC cells to gemcitabine treatment. Exosomes derived from the PANC-1 cell line (which is more prone to gemcitabine resistance) were shown to overexpress the Ephrin type-A receptor 2 (EphA2) compared with other less chemoresistant cell lines (MIA PaCa-2 and BxPC-3) [79]. When EphA2 expression was suppressed in PANC-1 cells, their ability to transmit exosome-mediated chemoresistance to MIA PaCa-2 and BxPC-3 cells was inhibited, suggesting the possible role of EphA2 in promoting the resistance of PANC-1 cells to gemcitabine treatment and the transmission of the same to other cells.

Regarding RNAs, many studies have shown how RNAs, especially non-coding RNAs contribute to PC development and progression. For instance, miR-301a-3p derived from hypoxic PC cells induced the differentiation of macrophages into the M2 phenotype and enhanced the metastatic competence of PC cells by activating the PTEN/PI3Kγ signalling pathway [80]. Similarly, exosomal LINC01133 was found to be overexpressed in PC and associated with disease progression and metastasis [81]. LINC01133 interacted with the enhancer of zeste homolog 2 (EZH2) to promote the trimethylation of histone H3 on lysine 27 (H3K27). This was achieved via the suppression of the axis inhibition protein 2 (AXIN2) and glycogen synthase kinase 3 (GSK-3) activities, resulting in the activation of β-catenin. This promoted an epithelial-mesenchymal transition (EMT). These and many other studies [82,83,84,85] have proven that exosomal RNAs can be associated with pancreatic malignancy.

## 6. Pancreatic Stellate Cell-Derived Exosomes and PCRD

Pancreatic stellate cells (PSCs) are stromal cells that are present around the earliest neoplastic lesions and around islets. PSCs are responsible for producing the collagenous stroma of PC and interact closely with cancer cells to drive cancer progression. Recent studies suggest that exosomes secreted by PSCs may play a major role in stroma-tumour cross-talk [86].

In this regard, exosomal miR-5703 was found to target CKLF Like MARVEL Transmembrane Domain Containing 4 (CMTM4) which is known to regulate the expression of the inhibitory programmed death-ligand 1 (PD-L1) and to stabilize the PD-L1 receptor in cancers. By binding to CMTM4, the PC-derived exosomal miR-5703 promoted the proliferation of PC cells via the PAK4-activated PI3K/Akt pathway [87]. Additionally, when PC cells internalized PSC-derived exosomes, this resulted in high miR-21 levels, which promoted cell migration, EMT, increased matrix metalloproteinase-2/9 activity and enhanced Ras/ERK signalling activity by increasing ERK1/2 and Akt phosphorylation in the PC cells [88]. Additionally, hypoxia-induced PSC-derived exosomal miRNAs—miR-4465 and miR-616-3p—targeted the tumour-suppressing phosphatase and tensin homolog (PTEN) and activated AKT signalling in PC cells, promoting proliferation, migration and invasion [89].

**Figure 4:**
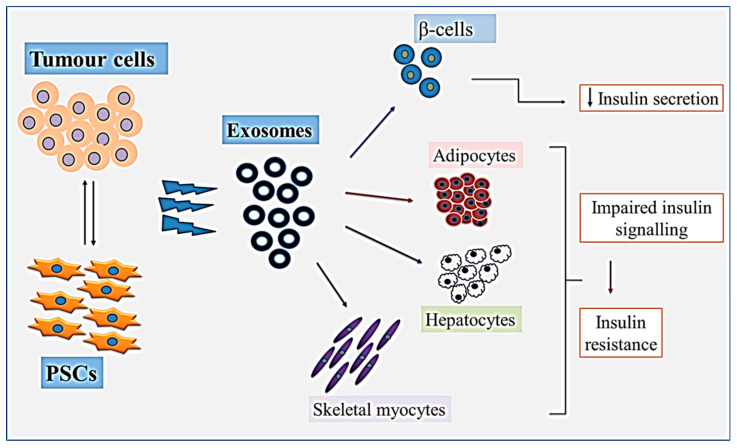
Interactions of pancreatic stellate cells (PSCs) and tumour cells lead to the release of exosomes which could be taken up by (i). β cells resulting in decreased insulin secretion, and (ii). adipocytes, hepatocytes and/or myocytes, could result in impaired insulin signalling and insulin resistance.

Of particular relevance to this review is a recent study that demonstrated how exosomes derived from co-cultures of mouse PSCs and PC cells caused mouse β cell dysfunction by decreasing the β cell proliferation and inhibiting their insulin secretion capacity in response to glucose stimulation, showing that exosomes secreted by PSCs and PC cells carry factors that negatively regulate the functions of islet cells [90] (Figure 4). Whether PSC-derived exosomes play a role in peripheral insulin resistance is yet to be determined. Nevertheless, it is possible that the exosomal cargo from both PC cells and stromal cells (PSCs) play a role in the pathogenesis of PCRD.

## 7. Other Biomarkers for PCRD

Studies have also identified some mechanisms and genomic markers for PCRD, although the localization of these biomarkers in exosomes has not yet been established. For example:

1. Nitric oxide (NO), which is overexpressed in PCRD, has diabetogenic effects, and the absence of specific fragments of NO correlates with the development of DM [91]. Hence, PCRD might be a result of the diabetogenic effects of tumour products, possibly acting via NO.
2. In a study aimed at identifying the mediators of PCRD by comparing the gene expression between PCRD patients and PC patients without DM, low levels of Kinesin Family Member 22 (KIF22) and glycogen Phosphorylase L (PYGL) were found to be associated with good survival outcomes for PC patients with DM and may be prognostic biomarkers for PCRD. Additionally, bioinformatic predictions revealed that KIF22, PYGL, Ribosomal Protein S27a (RPS27A), and ubiquitin A-52 residue ribosomal protein fusion product 1 (UBA52) could be involved in the pathogenesis of PCRD [92].

Further studies are required to determine whether the role of these markers in PCRD is exosome-based. Considering the stability, relevance, and reliability of exosome-based biomarkers, we recommend that in future studies, the functions of these biomarkers are examined to determine whether they are enhanced by exosomes.

## 8. Summary

Pancreatic cancer-related diabetes (PCRD) is a condition that identifies a specific subset of patients who may feasibly benefit from the screening strategies for pancreatic cancer. Understanding the pathogenesis of PCRD, particularly the role of pancreatic cancer-derived exosomal cargo in disrupting glucose homeostasis is an important first step when identifying the factors that could be used as biomarkers for early diagnosis, the prediction of response to therapy as well as prognosis. The role of PC-derived exosomal cargo components, particularly proteins and microRNAs, in PCRD, is becoming increasingly elucidated. Additionally, the exosomal factors involved in PCRD but not longstanding Type 2 diabetes have also been identified. Thus, there are some promising advances in this field that bode well for the development of clinical applications in the near future and could enable timely diagnosis and improved outcomes for patients with pancreatic cancer.

## Figures and Tables

**Table 1:** Differences between PCRD and T2DM.

S/N	PCRD	T2DM
1.	Patients develop DM despite preceding weight loss	Patients gain weight at the time of DM onset
2.	Occurs many months before the occurrence of cachexia (with greater than a 5% weight loss over 6 months)	Not associated with cachexia
3.	Occurs within 3–5 years before the clinical diagnosis of PC	Occurs with or without PC development
4.	Insulin levels are normal or low	Insulin levels are high and there is a marked insulin resistance
5.	Usually improves or is resolved following PC treatments, including the surgical resection of the tumour	Persistent DM accompanies PDAC patients with long-standing T2DM even after surgical resection
6.	Low levels of the glucose-dependent insulinotropic polypeptide (GIP) are evident	Variable levels of GIP
7.	Pancreatic polypeptide (PP) levels are low or absent	PP levels are high or normal
8.	Low glucagon levels	High glucagon levels
9.	Occurs at any age	Occurs mainly in adulthood

## Data Availability

Not applicable.
